# Metabolomic Analysis of Plant Hormone-Related Metabolites in *Medicago sativa* Under Low-Temperature Stress

**DOI:** 10.3390/molecules30163373

**Published:** 2025-08-13

**Authors:** Yue Zhao, Jie Wang, Chengti Xu, Yuanyuan Zhao, Xiuzhang Li, Jing Liu, Xiaojian Pu

**Affiliations:** 1 Academy of Animal Science and Veterinary Medicine, Qinghai University, Xining 810016, China; zhaoyue010306@163.com (Y.Z.); wangjie08142023@163.com (J.W.); xchti@163.com (C.X.); 18893147262@163.com (Y.Z.); xiuzhang11@163.com (X.L.); 2 Key Laboratory of Northwest Cultivated Land Conservation and Marginal Land Improvement Enterprises, Ministry of Agriculture and Rural Affairs, Delingha 817000, China; 3 College of Ecological Environment and Resources, Qinghai Minzu University, Xining 810007, China; 2009007@qhmu.edu.cn

**Keywords:** low-temperature stress, alfalfa, plant hormones, metabolomics

## Abstract

(1) Background: This study used the cold-tolerant cultivar “Daye No. 3” (DY) and the cold-sensitive cultivar “Longdong” (LD) as plant materials to study the metabolic changes in plant hormones in alfalfa (*Medicago sativa* L.) under cold stress. (2) Methods: The targeted quantitative detection of phytohormones in alfalfa was carried out by liquid chromatography–tandem mass spectrometry (LC-MS/MS) technology. Principal component analysis (PCA), orthogonal signal correction, and partial least squares discriminant analysis (OPLS-DA) were used to investigate sample classification and screen differential metabolites. (3) Results: The results showed that 17 differential metabolites were detected. Seven metabolites showed common changes in the two cultivars after low-temperature stress induction. The levels of tryptamine, N-jasmonoylisoleucine, trans-zeatin riboside, isopentenyladenine riboside, cis-zeatin riboside, and gibberellin A7 were decreased, while N6-isopentenyladenine levels increased. In addition, compared with the LD variety, DY had more metabolite changes in response to low-temperature stress. Abscisic acid and trans-zeatin were elevated, whereas IAA-alanine, dihydrozeatin riboside, and indole-3-carboxaldehyde showed reduced concentrations. Kyoto Encyclopedia of Genes and Genomes (KEGG) pathway enrichment analysis showed that differential plant hormones were more active in plant hormone signal transduction, zeatin biosynthesis, and tryptophan metabolism pathways. In addition, a total of 12 metabolites in these three pathways showed common changes under cold stress. (4) This study identified significant metabolomic differences between two alfalfa genotypes under stress. It highlighted key pathways and provided new insights into the metabolic changes of alfalfa under cold-stress conditions.

## 1. Introduction

Alfalfa (*Medicago sativa* L.) is a perennial leguminous forage crop that is widely cultivated and utilized worldwide, recognized for its high biomass, rich nutritional value, strong adaptability, and excellent palatability [1,2,3]. With the rapid development of the livestock industry, the demand for alfalfa in China has been increasing. However, the current production of high-quality alfalfa remains insufficient to meet this demand, making it crucial to enhance both the yield and quality of alfalfa [4]. Research indicates that within an average annual temperature range of 0–20 °C, there is a significant positive correlation between the yield and quality of alfalfa and environmental temperature [5]. Nevertheless, in the high-altitude regions of the Qinghai-Tibet Plateau, low temperatures significantly decrease the overwintering survival rate and fresh yield of alfalfa [6]. Low-temperature stress, as one of the major environmental factors limiting agricultural productivity, alters the lipid composition of cell membranes, affecting membrane fluidity, and can disrupt membrane integrity, leading to solute leakage. Additionally, it induces the accumulation of reactive oxygen species, exacerbating oxidative damage, which severely impairs plant growth and development [7,8,9].

Plants have evolved intricate mechanisms to defend against cold stress. Cold acclimation refers to the process by which plants adapt to low temperatures, thereby enhancing their freezing tolerance through metabolic adjustments at non-freezing temperatures [10]. This process encompasses a series of complex biochemical, molecular, and metabolic changes [11], accompanied by significant alterations in the expression of multiple genes, mediated by various plant hormones [12]. Abscisic acid (ABA) is a crucial plant hormone that plays a vital role in regulating plant growth [13] and stress resistance [14]. On one hand, low-temperature stress can elevate the endogenous levels of ABA in plants [15]. Conversely, exogenous ABA treatment can improve the cold resistance of plants and plays a beneficial role in maintaining the stability and normal function of cell membranes [16,17]. Additionally, other plant hormones such as auxin [18], jasmonic acid [19], cytokinin [20], and gibberellin [21] collectively contribute to regulating plant responses to low-temperature stress.

In recent years, researchers have systematically investigated the dynamic response mechanisms of alfalfa to cold stress at the transcriptome level. Studies indicate that differentially expressed genes (DEGs) are significantly enriched in plant hormone signal transduction pathways [22]. Furthermore, our previous research identified 550 auxin-related genes, 1914 abscisic acid-related genes, 242 cytokinin-related genes, and 104 gibberellin-related genes that are responsive to cold stress [23]. Notably, current investigations into plant hormones at the metabolomic level in response to low temperatures are relatively scarce, resulting in a limited understanding of their roles in the overall regulation of plant responses to cold. Therefore, this study employs targeted metabolomics technology to examine the metabolic changes in alfalfa under low-temperature stress, aiming to elucidate the intricate relationship between plant hormones and cold tolerance, as well as to identify potential cold-resistant metabolic markers. This research provides new insights and approaches for enhancing the cold tolerance of alfalfa, which has significant application value for advancing stress-resistant breeding practices in forage crops.

## 2. Results

### 2.1. Overall Sample Quality Control and PCA Analysis

**Figure 1:**
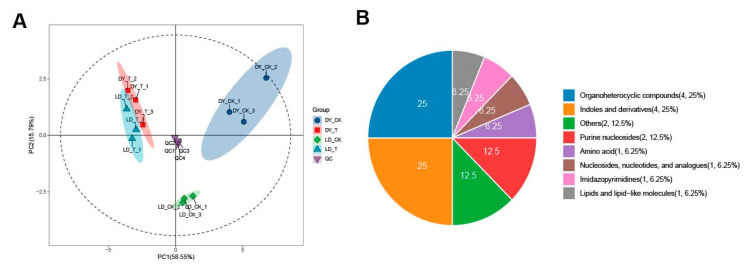
Metabolomics profiles of different treatments. (**A**) PCA analysis of overall samples. (**B**) Classification and annotation of differential metabolites. The first number in parentheses indicates the number of metabolites in the category, and the second indicates their proportion of the total identified metabolites.

The results of the principal component analysis (PCA) indicate significant differences in the metabolite profiles among different groups, with the first two principal components, PC1 and PC2, accounting for 58.55% and 15.79% of the variance, respectively (Figure 1A). The control groups of both varieties (LD_CK and DY_CK) exhibit a good degree of internal clustering, and there is a notable trend of separation between the groups. Furthermore, significant separation trends were observed between the control and treated groups within each variety (LD_CK vs. LD_T; DY_CK vs. DY_T). The treatment groups DY_T and LD_T not only show good internal clustering but also exhibit a significant trend of clustering between the groups, indicating that these components effectively distinguish different samples.

### 2.2. OPLS-DA, and Permutation Test of Different Treatment Groups

**Figure 2:**
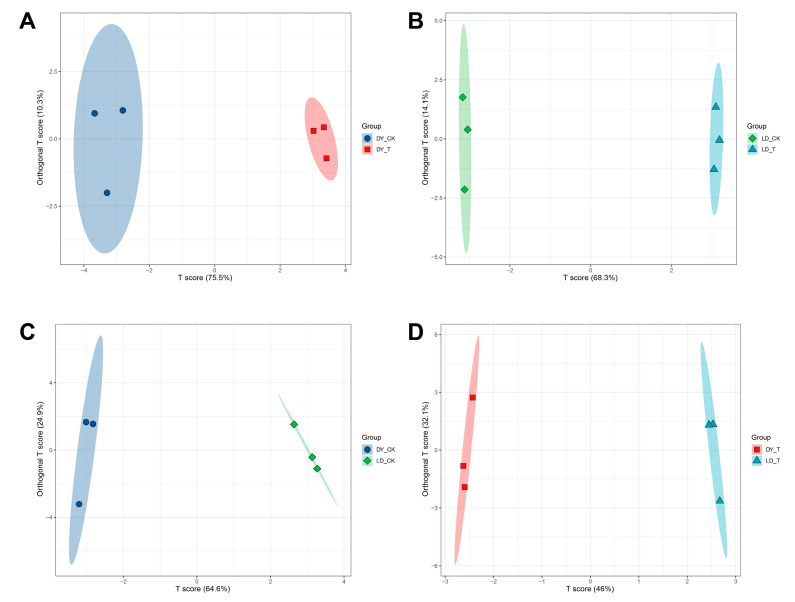
OPLS-DA score plots. (**A**) DY_CK vs. DY_T, (**B**) LD_CK vs. LD_T, (**C**) DY_CK vs. LD_CK, (**D**) DY_T vs. LD_T.

Orthogonal partial least squares discriminant analysis (OPLS-DA) is a regression modeling method that analyzes multiple dependent variables against multiple independent variables [24]. To further demonstrate the differences between groups and identify distinct metabolites, the metabolite contents were normalized, followed by orthogonal partial least squares discriminant analysis to obtain OPLS-DA score plots. In this model, R^2^X (cum) and R^2^Y (cum) represent the interpretation rates of the constructed model for the X and Y matrices, respectively, while Q^2^ indicates the predictive ability of the model. The OPLS-DA model was used to compare the metabolite compositions of DY_CK vs. DY_T: the contribution rates of the first and second principal components were 75.5% and 10.3%, respectively, with R^2^X = 0.858, R^2^Y = 0.993, and Q^2^ = 0.974 (Table 1, Figure 2A); LD_CK vs. LD_T: the contribution rates of the first and second principal components were 68.3% and 14.1%, respectively, with R^2^X = 0.944, R^2^Y = 1, and Q^2^ = 0.997 (Table 1, Figure 2B); DY_CK vs. LD_CK: the contribution rates of the first and second principal components were 64.6% and 24.9%, respectively, with R^2^X = 0.895, R^2^Y = 0.995, and Q^2^ = 0.976 (Table 1, Figure 2C); and DY_T vs. LD_T: the contribution rates of the first and second principal components were 46% and 32.1%, respectively, with R^2^X = 0.881, R^2^Y = 0.999, and Q^2^ = 0.965 (Table 1, Figure 2D). All pairwise comparisons showed high R^2^X, R^2^Y, and Q^2^ values, indicating that these analyses are reproducible, reliable, and suitable for screening differential metabolites. To evaluate the robustness of the OPLS-DA models and exclude overfitting, 200-time permutation tests were conducted for each comparison group. The results showed that the Q^2^ intercepts of all the models were less than 0, indicating no overfitting and good predictive ability (Figure S1). These findings confirm that the constructed OPLS-DA models are suitable for identifying differential metabolites under cold stress.

### 2.3. Screening of Differential Metabolites

A total of 17 significant differential metabolites were detected in the sample, categorized into eight classes. These include four types of indoles and derivatives (25%), four types of organic heterocyclic compounds (25%), two types of purine nucleosides (12.5%), one type of organic acid and its derivatives, one type of nucleoside, nucleotide, and analogue (6.25%), one type of lipid and lipid-like molecule (6.25%), one type of imidazopyrimidine (6.25%), one type of amino acid (6.25%), and two others (12.5%) (Figure 1B). In the DY_CK vs. DY_T comparison, twelve metabolites showed significant changes, with three increased and nine decreased. The LD_CK vs. LD_T comparison showed more conservative changes: eight metabolites exhibited significant changes, with one increased and seven decreased. In the DY_CK vs. LD_CK comparison, six metabolites showed significant changes, with five increased and one decreased. The comparison between treated groups (DY_T vs. LD_T) showed significant changes in five metabolites, with four increased and one decreased (Table 2).

### 2.4. Importance Analysis of Differential Metabolites

**Figure 3:**
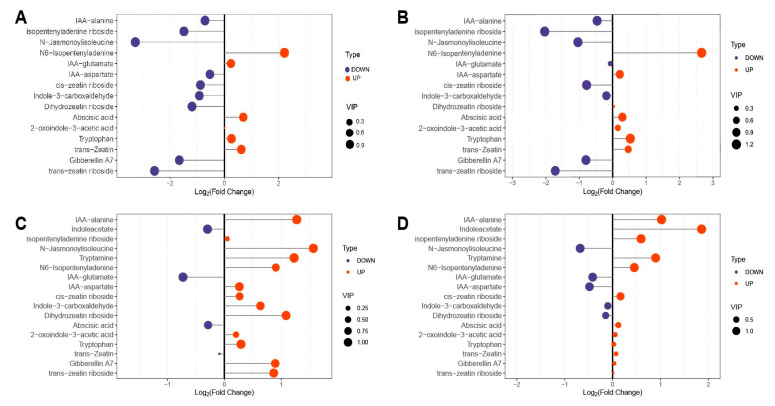
Analysis of the importance of differential metabolites. (**A**) DY_CK vs. DY_T, (**B**) LD_CK vs. LD_T, (**C**) DY_CK vs. LD_CK, (**D**) DY_T vs. LD_T.

Under low-temperature treatment, the VIP values of tryptamine, N6-isopentenyladenine, N-jasmonoylisoleucine, and isopentenyladenine riboside were all greater than 1.0, indicating that these four metabolites are of the highest importance. The accumulation and depletion patterns of differentially abundant metabolites were generally consistent between the two alfalfa varieties, suggesting similar physiological mechanisms of cold stress resistance (Figure 3A,B). Under the same treatment, the levels of some differentially abundant metabolites differed significantly between the two alfalfa varieties. Specifically, indoleacetate, abscisic acid, and trans-zeatin were significantly increased following low-temperature treatment. Conversely, N-jasmonoylisoleucine, indole-3-carboxaldehyde, and dihydrozeatin riboside were significantly decreased after low-temperature treatment (Figure 3C,D).

### 2.5. Cluster Analysis

**Figure 4:**
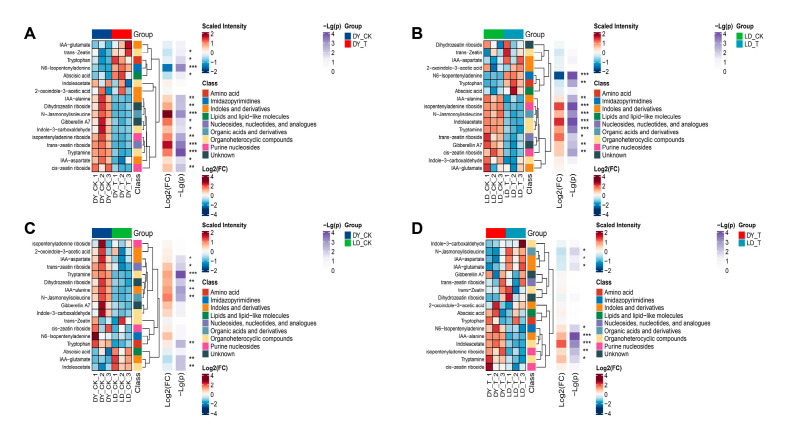
Heatmap of clustering analysis of metabolites. Red indicates higher levels, and blue indicates lower levels. Asterisks indicate significance levels: * *p*< 0.05, ** *p*< 0.01, *** *p*< 0.001. (**A**) DY_CK vs. DY_T, (**B**) LD_CK vs. LD_T, (**C**) DY_CK vs. LD_CK, (**D**) DY_T vs. LD_T.

Clustering clearly separates the DY_T group and DY_CK group, as well as the LD_T and LD_CK groups, showing that low-temperature treatment significantly altered metabolite levels. Compared to the control group, in the DY_T group, the levels of ABA, N6-isopentenyladenine, trans-zeatin, and tryptophan increased and clustered together, with N6-isopentenyladenine showing a particularly significant increase. In contrast, the levels of 10 metabolites decreased, with N-jasmonoylisoleucine, trans-zeatin riboside, and tryptamine significantly reduced (Figure 4A). The changes in the LD variety were relatively minor compared to the control group. In the LD_T group, the levels of N6-isopentenyladenine and tryptophan increased, while the levels of 8 metabolites decreased. Notably, isopentenyladenine riboside, N-jasmonoylisoleucine, indoleacetate, and tryptamine were significantly reduced (Figure 4B).

Clustering clearly separates the DY_CK and LD_CK groups, as well as the DY_T and LD_T groups, highlighting metabolic differences between the two varieties under both control and low-temperature conditions. Compared to the DY_CK group, the LD_CK group showed decreased levels of seven metabolites, with tryptamine significantly reduced, while IAA-glutamate and indoleacetate were increased (Figure 4C). In comparison to the DY_T group, five metabolites were decreased in the LD_T group, with IAA-alanine and indoleacetate significantly reduced. Meanwhile, three metabolites, including N-jasmonoylisoleucine, were increased (Figure 4D).

### 2.6. Correlation Analysis

**Figure 5:**
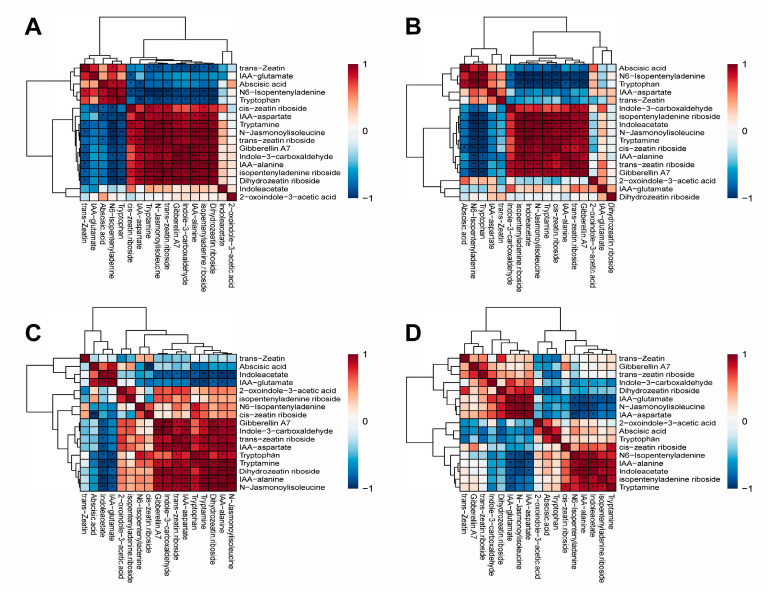
Correlation analysis of metabolites. Positive correlations are indicated in red, while negative correlations are indicated in blue. Asterisks indicate significance levels: * *p*< 0.05, ** *p*< 0.01, *** *p*< 0.001. (**A**) DY_CK vs. DY_T, (**B**) LD_CK vs. LD_T, (**C**) DY_CK vs. LD_CK, (**D**) DY_T vs. LD_T.

Under low-temperature treatment, both the DY_CK vs. DY_T and LD_CK vs. LD_T comparisons exhibited similar metabolite correlation patterns. Specifically, indole-3-carboxaldehyde, isopentenyladenine riboside, tryptamine, cis-zeatin riboside, IAA-alanine, trans-zeatin riboside, gibberellin A7, and N-jasmonoylisoleucine showed positive correlations, while tryptophan, N6-isopentenyladenine, and abscisic acid exhibited negative correlations. Notable differences were also observed between the two varieties. In the DY group, IAA-aspartate and dihydrozeatin riboside were among the positively correlated metabolites, whereas IAA-glutamate and trans-zeatin were among the negatively correlated metabolites. By comparison, in the LD group, indoleacetate was positively correlated, while IAA-aspartate was negatively correlated (Figure 5A,B).

Under non-low-temperature treatment, gibberellin A7, indole-3-carboxaldehyde, trans-zeatin riboside, IAA-aspartate, tryptophan, dihydrozeatin riboside, and N-jasmonoylisoleucine exhibit a positive correlation. Under low-temperature treatment, cis-zeatin riboside, N6-isopentenyladenine, indoleacetate, and isopentenyladenine riboside show a positive correlation (Figure 5C). Under non-low-temperature treatment, trans-zeatin, abscisic acid, and indoleacetate demonstrate a negative correlation. Under low-temperature treatment, indole-3-carboxaldehyde, dihydrozeatin riboside, N-jasmonoylisoleucine, and IAA-aspartate exhibit a negative correlation (Figure 5D). The two varieties exhibited similar patterns under different treatment conditions; tryptamine and IAA-alanine demonstrated a positive correlation, and IAA-glutamate showed a negative correlation (Figure 5C,D).

### 2.7. KEGG Analysis

**Figure 6:**
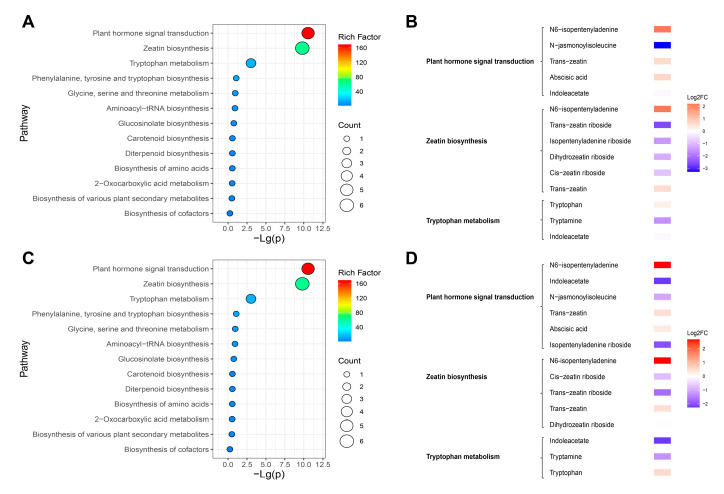
KEGG enrichment analysis. (**A**) DY_CK vs. DY_T enrichment analysis, (**B**) Alterations in metabolites within metabolic pathways between DY_CK and DY_T, (**C**) LD_CK vs. LD_T enrichment analysis, (**D**) Alterations in metabolites within metabolic pathways between LD_CK and LD_T.

Differential metabolites are primarily enriched in three metabolic pathways under the cold-stress treatment, including plant hormone signal transduction, biosynthesis of zeatin, and tryptophan metabolism (Figure 6). In the plant hormone signal transduction pathway, both alfalfa cultivars exhibited decreased auxin levels in tryptophan metabolism and increased cytokinin levels in the biosynthesis of zeatin after cold stress. Additionally, abscisic acid levels increased in the biosynthesis of carotenoids, and JA-ile levels decreased in α-linolenic acid metabolism. Auxin in tryptophan metabolism, cytokinins in zeatin biosynthesis, and abscisic acid in carotenoid biosynthesis decreased in the DY_CK vs. LD_CK group but increased in the DY_T vs. LD_T group. JA-ile in α-linolenic acid metabolism increased in the DY_CK vs. LD_CK group, while it decreased in the DY_T vs. LD_T group (Tables S3 and S4).

In the biosynthetic pathway of zeatin, two alfalfa species exhibited decreased levels of isopentenyl-adenosine, trans-zeatin riboside, and cis-zeatin riboside following cold stress, while isopentenyladenine and trans-zeatin showed increased levels. Dihydrozeatin riboside decreased in the DY_CK vs. DY_T group but increased in the LD_CK vs. LD_T group (Tables S2 and S5). Isopentenyl-adenosine, trans-zeatin riboside, cis-zeatin riboside, and isopentenyladenine showed higher levels in the DY_CK vs. LD_CK group and DY_T vs. LD_T group, while dihydrozeatin riboside was decreased in the DY_T vs. LD_T group but increased in the DY_CK vs. LD_CK group. Trans-zeatin increased in the DY_T vs. LD_T group but decreased in the DY_CK vs. LD_CK group (Tables S3 and S4).

In the metabolism of tryptophan, the two types of alfalfa exhibited a decrease in tryptamine and indoleacetate following cold stress, while tryptophan was increased. Tryptophan and tryptamine levels were higher in both the DY_CK. vs. LD_CK and DY_T. vs. LD_T comparisons, whereas indoleacetate increased in the DY_T. vs. LD_T group but decreased in the DY_CK. vs. LD_CK group (Tables S3 and S4).

## 3. Discussion

Low temperature is a major abiotic stress factor limiting plant growth, development, and productivity [25]. Changes in plant hormones play a crucial role in their tolerance mechanisms to low-temperature stress [26]. Among them, ABA (abscisic acid) has important functions in plant responses to drought and low-temperature stress [27]. Studies have shown that plant hormone signal transduction pathways are drastically enriched under drought stress, and ABA significantly accumulates in metabolites [28]. In this study, we observed that the cold-tolerant cultivar, DY, exhibited significantly higher ABA levels than the sensitive cultivar, LD, under low-temperature stress, suggesting a potential role of ABA in cold stress response. Similarly, previous studies on *Zanthoxylum bungeanum* also revealed that the cold variety exhibited significantly higher ABA levels compared to the cold-sensitive one, and the rapid ABA increase during cold stress may help activate cold-responsive mechanisms and improve cold tolerance [29]. This finding supports the pivotal regulatory role of ABA in plant responses to cold stress. However, other studies have reported that cold-sensitive soybean cultivars exhibit higher ABA levels than cold-tolerant ones under low-temperature stress [30]. Such discrepancies may be attributed to factors such as cultivar characteristics, sampling time points, and stress intensity, indicating that the role of ABA in low-temperature response remains to be fully elucidated. Furthermore, this study did not directly evaluate phenotypic traits, limiting the ability to fully link ABA accumulation to cold tolerance. However, previous work from our group showed that the cold-tolerant genotype exhibits higher aboveground biomass under cold stress, which may partly relate to its elevated ABA levels; this is further supported by transcriptomic results, where ABA receptor genes such as PYL8 and core signaling components like SnRK2 were significantly upregulated under cold stress, suggesting activation of the ABA signaling pathway and a potential link between elevated ABA levels and enhanced cold tolerance [23]. Future studies integrating metabolite and phenotypic analyses are needed to clarify this relationship and the role of ABA in cold tolerance.

The accumulation of plant hormones and metabolites may help establish a primed state, thereby promoting better tolerance to subsequent stresses [31]. In this study, seven metabolites showed common changes in both cultivars after low-temperature stress induction, among which tryptamine, N-jasmonoylisoleucine (JA-ile), trans-zeatin riboside, isopentenyladenine riboside, cis–zeatin riboside, and gibberellin A7 were decreased, while N6-isopentenyladenine was increased. This may represent common changes induced by low-temperature stress. Hierarchical clustering and correlation analysis highlighted N6-isopentenyladenine, JA-ile, and tryptamine as key metabolites involved in the cold stress response. N6-isopentenyladenine, a cytokinin derivative, significantly increased after cold treatment, suggesting plants may enhance growth regulation to maintain adaptability under cold stress. Similar cytokinin dynamics have been reported in Arabidopsis and Secale cereale under cold stress. In Arabidopsis, cold stress significantly reduced levels of highly active cytokinins such as trans-zeatin and its riboside forms, while increasing less active precursors like isopentenyladenine, potentially as a strategy to downregulate growth while preserving basal cytokinin signaling [32]. Likewise, in rye, cold stress led to a marked decrease in active CKs and an increase in trans-zeatin riboside, the transport form of CK, suggesting a role in stress adaptation and long-distance signaling [33]. In mango, cold-induced upregulation of cytokinin signaling genes such as AHK and ARR-B indicated enhanced CK signal transduction, which may contribute to improved cold tolerance [34]. These findings highlight the pivotal role of cytokinin metabolism and transport in coordinating growth inhibition and facilitating cold acclimation. In contrast, JA-ile and tryptamine levels significantly decreased, indicating that plants may conserve energy and redirect resources by suppressing jasmonate and auxin pathways during cold exposure. Correlation analysis further revealed a negative correlation between N6-isopentenyladenine and JA-ile, and a positive correlation between JA-ile and tryptamine. These results suggest potential antagonism between cytokinins and jasmonates and coordination between jasmonate and auxin metabolism. These findings underscore the complex interplay of hormone-related metabolites in regulating cold stress responses.

Compared with the LD cultivar, DY exhibited more metabolic changes in response to low-temperature stress, including increased abscisic acid and trans-zeatin levels, and decreased IAA-alanine, dihydrozeatin riboside, and indole-3-carboxaldehyde. These metabolic changes may contribute to its enhanced cold resistance. Notably, indoleacetate decreased in LD but showed no significant change in DY, suggesting that auxin suppression may play a greater role in cold-induced growth inhibition in the sensitive cultivar. Hierarchical clustering further revealed that DY primarily reduced trans-zeatin riboside, while LD showed a broader decrease in both cytokinin- and auxin-related metabolites (isopentenyladenine riboside and indoleacetate). Under non-stress conditions, the DY cultivar exhibited significantly higher levels of metabolites, such as tryptamine, JA-ile, trans-zeatin riboside, IAA-alanine, and dihydrozeatin riboside, compared to the LD cultivar, whereas LD showed higher levels of IAA-glutamate. This suggests that the DY maintains elevated levels of auxin-, cytokinin-, and jasmonic acid-related metabolites under normal conditions, which may contribute to higher growth activity and an inherent ability to respond to stress. Following cold treatment, DY still maintained higher levels of tryptamine, isopentenyladenine riboside, IAA-alanine, and indoleacetate, while LD showed higher levels of JA-ile. These results indicate that DY may maintain or enhance the accumulation of certain hormone-related metabolites under cold stress, potentially contributing to its cold tolerance. Under cold stress, the sensitive cultivar, LD, exhibited elevated levels of JA-ile compared to DY, indicative of a strong defense activation. This pattern parallels the response observed in rice, where leaf-targeted cold stress induced JA and JA-ile accumulation may have contributed to enhanced cold tolerance by alleviating the adverse effects of stress on photosynthetic activity [35]. In contrast, hierarchical clustering analysis showed that IAA-alanine and indoleacetate were significantly reduced in LD under cold stress, suggesting that the auxin metabolism pathway was inhibited, possibly leading to greater growth suppression. These results indicate that DY sustains hormonal balance under cold stress to coordinate growth and defense, whereas LD prioritizes jasmonate-mediated responses, potentially at the cost of growth. This reflects distinct adaptive strategies of the two cultivars in coping with cold stress.

In the intergroup comparison, we found that the two alfalfa cultivars had overlapping and significantly enriched metabolic pathways, mainly including plant hormone signal transduction, zeatin biosynthesis, and tryptophan metabolism pathways. The differential metabolites involved in these pathways may serve as potential biomarkers for distinguishing cold-tolerant cultivars. Studies have shown that plant hormone signal transduction plays an important role in alfalfa’s response to stress [36], which is consistent with the results of this study. Additionally, previous research indicated that tryptophan metabolism levels increase in plants under drought stress, and tryptophan may play a major role in improving plant drought resistance [37]. This study also observed significant enrichment of the tryptophan metabolism pathway, suggesting its potential importance in alfalfa’s low-temperature response. Notably, among the three enriched pathways, the zeatin biosynthesis pathway showed the most significant intergroup differences. Studies have shown that the zeatin biosynthesis pathway plays an important role in plant resistance to abiotic stresses [38,39]. However, a limitation of this study is that samples were collected at a single time point (24 h) after cold treatment. Given the dynamic nature of metabolic changes, future research should incorporate multiple time points to better characterize the temporal patterns of hormone regulation during cold exposure and recovery. Furthermore, integrating transcriptomic or gene expression data related to key biosynthetic and signaling genes would provide deeper mechanistic insight into the observed metabolic differences.

## 4. Materials and Methods

### 4.1. Test Materials and Cold Treatment

The experimental materials selected for this study were the cold-resistant cultivar “Daye No. 3 (DY)” and the cold-sensitive cultivar “Longdong (LD)” of Medicago sativa. The seeds were provided by the College of Animal Science and Veterinary Medicine, Qinghai University. Approximately 15 seeds of Medicago sativa were evenly sown in seedling bowls with a diameter of 15 cm and a height of 14 cm. The growth medium used was nutrient-rich soil. After germination, the plant density was reduced to 9 plants per bowl, and cultivation continued. The seedlings were grown in a temperature-controlled incubator under a 16 h light and 8 h dark cycle, with a light period at 23 °C, a dark period at 18 °C, and a light intensity of 6000 lx. After 18 days of seedling cultivation, the cold treatment was conducted. The pre-cultivated DY and LD seedlings were divided into a control group (CK) and a treatment group (T). The CK group continued to be cultivated under the previous conditions, while the T group was subjected to a cold acclimation treatment, consisting of three cycles of 12 h at 4 °C followed by recovery at 23 °C in a temperature-controlled incubator. After these cycles, the plants were maintained at 4 °C to induce cold stress. This stepwise treatment was designed to gradually activate cold-responsive pathways. After a 24 h cold treatment at 4 °C, leaf samples were collected for hormone metabolite determination. Leaves from different replicates of the same treatment were cut, thoroughly mixed, rapidly processed in liquid nitrogen, and stored at −80 °C for subsequent measurement of relevant indicators. Each treatment had three replicates.

### 4.2. Metabolite Extraction

Each sample was ground under liquid nitrogen conditions, with a weight of 40 mg. Subsequently, 1000 μL of pre-cooled 50% acetonitrile was added along with two medium-sized steel beads. The samples were homogenized in a tissue disruptor at low temperature for 5 min, followed by ultrasound treatment in an ice bath for 3 min. Extraction was conducted in a refrigerator at 4 °C for 30 min, after which the samples were centrifuged at 16,000× *g* for 10 min at 4 °C to collect the supernatant. The supernatant was purified using solid-phase extraction (SPE) columns, and the eluate was collected for vacuum drying. The samples were then re-dissolved in 50 μL of pre-cooled 50% acetonitrile solution, subjected to ultrasound in an ice bath for 3 min, and centrifuged at 20,000× *g* for 15 min at 4 °C to obtain the supernatant for mass spectrometry analysis.

### 4.3. LC-MS/MS Analysis

The samples were separated using a Nexera X2 LC-30AD ultra-high-performance liquid chromatography system (Shimadzu, Kyoto, Japan). The mobile phase consisted of A: 0.1% formic acid aqueous solution and B: 0.1% formic acid in acetonitrile. The samples were placed in an automatic sampler at 4 °C, with a column temperature of 50 °C and a flow rate of 300 μL/min, and an injection volume of 10 μL. The relevant liquid chromatography gradient was as follows: from 0 to 5 min, B was maintained at 5%; from 5 to 15 min, B linearly increased from 5% to 80%; from 15 to 16 min, B linearly increased from 80% to 100%; from 16 to 17.5 min, B was maintained at 100%; from 17.5 to 17.7 min, B linearly decreased from 100% to 5%; and from 17.7 to 20 min, B was maintained at 5%. A QC sample was set up after a certain number of experimental samples in the sample queue to detect and evaluate the stability and reproducibility of the system; a mixture of standard plant hormone metabolites was included in the sample queue for chromatographic retention time calibration.

Mass spectrometry analysis was conducted using the 5500 QTRAP mass spectrometer (AB SCIEX) in both positive and negative ion modes. The parameters for electrospray ionization (ESI) were as follows: source temperature 550 °C, ion source gas1 (GAS1): 40 psi, ion source gas2 (GAS2): 50 psi, curtain gas (CUR): 35 psi, positive ion spray voltage floating (ISVF) 5500 V, and negative ion spray voltage floating (ISVF) −4500 V. The multiple reaction monitoring (MRM) mode was employed to detect the target ion pairs (Table S1).

### 4.4. Statistical Analysis

The multivariate statistical analysis of the data included grouped principal component analysis (PCA), partial least squares discriminant analysis (PLS-DA), PLS-DA model validation, orthogonal partial least squares discriminant analysis (OPLS-DA), and OPLS-DA model validation. To validate the OPLS-DA models and assess their robustness, 200-time permutation tests were performed. The model was considered reliable if the Q^2^ intercept was less than 0 or if the permuted Q^2^ value was consistently lower than the original Q^2^. All the groups met these criteria, confirming that the OPLS-DA models were not overfitted and had good predictive power. For PCA, the data were subjected to UV (unit variance scaling) processing using R (base package, 3.5.1) software. For OPLS-DA, after log2 transformation of the raw data, the data were centered using R (Metabo Analyst R, 1.0.1) software.

Based on the OPLS-DA dimensionality reduction method, the variable importance in projection (VIP) and fold change (FC) were calculated to determine the fold differences between groups. Additionally, the intensity of each metabolite component’s content and its explanatory power regarding sample classification discrimination were measured to aid in metabolite screening. Metabolite molecules were considered statistically significant when the *p*-value was <0.05. The Metabo Analyst software package (metaboanalyst.ca, 6.0) was used for functional pathway enrichment and topological analysis of the screened differential metabolites. The KEGG Mapper visualization tool was employed to explore the enriched pathways and obtain differential metabolite and pathway maps.

## 5. Conclusions

This study employed metabolomic analysis to evaluate the metabolic responses of the cold-sensitive Longdong and cold-tolerant Daye No.3 alfalfa (*Medicago sativa*) cultivars under low-temperature stress. The two cultivars exhibited both shared and distinct metabolic adjustments, particularly in hormone-related pathways. DY maintained higher baseline and post-stress levels of auxin- and cytokinin-related metabolites (e.g., tryptamine, IAA-alanine, indoleacetate, and isopentenyladenine riboside), suggesting a balanced strategy to sustain growth while coping with stress. In contrast, LD showed elevated jasmonoylisoleucine levels, indicative of a defense-oriented response potentially at the cost of growth. Additionally, plant hormone signal transduction, zeatin biosynthesis, and tryptophan metabolism pathways were significantly enriched in both cultivars, and the differential metabolites in these pathways could serve as potential biomarkers for cold tolerance in alfalfa seedlings. This study preliminarily revealed the metabolic response mechanisms and key metabolic pathways of alfalfa under low-temperature stress, providing a theoretical basis for further understanding its cold-resistant molecular mechanisms and scientific support for improving alfalfa cold-resistant breeding through metabolic marker screening.

## Figures and Tables

**Table 1:** Evaluation parameters of OPLS-DA models for comparison groups.

Group	R^2^X (cum)	R^2^Y (cum)	Q^2^ (cum)
DY_CK. vs. DY_T	0.858	0.993	0.974
LD_CK. vs. LD_T	0.944	1	0.997
DY_CK. vs. LD_CK	0.895	0.995	0.976
DY_T. vs. LD_T	0.881	0.999	0.965

**Table 2:** Changes in the number of differential metabolites under different treatments.

Group	Sig.	Increased	Decreased
DY_CK vs. DY_T	12	3	9
LD_CK vs. LD_T	8	1	7
DY_CK vs. LD_CK	6	5	1
DY_T vs. LD_T	5	4	1

## Data Availability

The original contributions presented in this study are included in the article/Supplementary Materials. Further inquiries can be directed to the corresponding author.

## Supplementary Materials

The following supporting information can be downloaded at: https://www.mdpi.com/article/10.3390/molecules30163373/s1, Figure S1: OPLS-DA Permutation Test; Table S1: Metabolite ion peak; Table S2: DY_CK. vs. DY_T KEGG enriched; Table S3: DY_CK. vs. LD_CK KEGG enriched; Table S4: DY_T. vs. LD_T KEGG enriched; Table S5: LD_CK. vs. LD_T KEGG enriched.
